# Editorial: Neutrophil death regulation in critical illness

**DOI:** 10.3389/fimmu.2023.1181056

**Published:** 2023-03-15

**Authors:** Jia-feng Wang, Bing-wei Sun, John C. Marshall

**Affiliations:** ^1^ Faculty of Anesthesiology, Changhai Hospital, The Naval Medical University, Shanghai, China; ^2^ Department of Burns and Plastic Surgery, Affiliated Suzhou Hospital of Nanjing Medical University, Suzhou, China; ^3^ Keenan Research Centre for Biomedical Science, Li Ka Shing Knowledge Institute, Toronto, ON, Canada; ^4^ Department of Surgery, St Michael’s Hospital, Toronto, ON, Canada; ^5^ Department of Critical Care Medicine, St Michael’s Hospital, Toronto, ON, Canada; ^6^ Institute of Medical Science, Temerty Faculty of Medicine, University of Toronto, Toronto, ON, Canada

**Keywords:** neutrophils, critical illness, cell death, inflammation, sepsis

Neutrophils are the most abundant white blood cells in peripheral blood, and the first line of defense against bacterial infection. They also contribute to organ injury in critical illness, in disorders such as sepsis, trauma, shock and acute pancreatitis (1). In response to pathogen-associated molecular patterns (PAMPs) or danger-associated molecular patterns (DAMPs), spontaneous neutrophil apoptosis is inhibited and cell survival prolonged. Dysregulation of neutrophil death is associated with impaired bactericidal capacity and an amplified inflammatory response, both of which lead to organ injury (2). In this series, we summarize recent experimental findings on:

- Dysregulation of neutrophil death in organ injury
- Neutrophil heterogeneity in a variety of diseases
- Neutrophil dysregulation in COVID-19

## Dysregulation of neutrophil death in organ injury

Delayed apoptosis of neutrophils and reduced neutrophil clearance contribute to lung injury in sepsis. A novel role for PD-L1 has been revealed in regulating the activity of PI3K/Akt pathway (3). In this topic, Zhu et al. reported that PD-L1/PI3K/Akt pathway regulated autophagy and the release of neutrophil extracellular traps (NETs). PD-L1 depletion in neutrophils resulted in attenuated lung injury and reduced NETs in the lung. The inhibition of NET release induced by PD-L1 knockout was reversed by antagonism of PI3K using wortmannin. LPS-induced lung injury could be attenuated by anti-PD-L1 antibody, suggesting that PD-L1 might be a promising target for treatment of lung injury induced by sepsis.

Zhu et al. also reviewed current knowledge on the dysregulation of neutrophil death in sepsis. Phagocytosis of bacteria can induce neutrophil apoptosis and necrosis, and the bacteria could be diminished by the lytic enzymes and reactive oxygen species (ROS). But the necrotic neutrophils might also release DAMPs and so exacerbate organ injury. The pathways overlap, and the processes including necroptosis, apoptosis, and pyroptosis have been called PANoptosis, reflecting a common role in coordinated cell death. It remains unclear which type of death neutrophils might undergo during sepsis.

Zhang et al. summarized the role of extracellular traps (ETs) in ischemic reperfusion injury. Most of the ETs are produced by neutrophils and called NETs. But ETs can also be released by the monocytes, macrophages, mast cells, eosinophils and basophils. The related pathways involved in NETosis have also been summarized. ET formation has been identified in ischemia reperfusion injury in the liver, kidney, intestine, lung, brain, heart, limb and even skin. Ischemia reperfusion injury may also lead to tumor recurrence and metastasis *via* NET formation.

Wan et al. performed a bibliometric and visual analysis of studies on NETs. The number of papers related to NETs has been increasing from 2004 to 2021 year by year. The countries, institutes and authors with the most publications are summarized. The most popular keywords of the publications related to NETs has been changed from “phagocytosis and antimicrobial peptide” to “stroke, citrullinated histone, cytokines storm, and COVID-19” recently.

## Neutrophil heterogeneity in diseases

Popularization of the use of single cell sequencing has revealed the heterogeneity of cells, including neutrophils, in a variety of diseases (4). Chen et al. investigated the differences between neutrophils from the neonatal umbilical cord blood (UCB) and those from healthy adults. The neutrophils from the UCB could be classified into immature and mature subsets according to the scRNA-seq. The maturation state of neutrophils correlated with the cell cycles. The neutrophils at G2 and G3 stages could predict the prognosis of diseases, such as sepsis, inflammation and tumor. The transcriptional characteristics differentiated the UCB neutrophils from those from healthy adults, and several transcription factors were identified to be potentially related to the regulation of neutrophil apoptosis.

Trzeciak et al. identified heterogeneity of C1q expression in neutrophils from septic patients. They subtyped neutrophils into CD49c^high^ or CD49c^low^ subsets. RNAseq data suggested that CD49c^high^ neutrophils were correlated with genes in the complement cascade, and the most differentially expressed complement-related genes were those encoding the C1q protein. C1q expression might differentiate surviving patients from those who died of sepsis. Its secretion was enhanced in apoptotic neutrophils and appears to be an “eat me” signal to promote neutrophil clearance.

Lavie et al. reported a new population of CD66b^+^ giant phagocytes (Gφ) in human carotid atherosclerotic plaques. It had been shown that some of the neutrophils cultured *in vitro* might have prolonged life span and become giant phagocytes with a single nucleus (5). Significant heterogeneity was present regarding the presence of CD66b^+^ neutrophils, which were negatively correlated with lipid level and positively correlated with the nitrosative stress marker 3-nitrotyrosine (3-NT) in the plaques.

## Neutrophil dysregulation in COVID-19

The COVID-19 pandemic resulted in a large number of death across the world and factors associated with an increased risk of death have been widely investigated. Qiu et al. studied a cohort of 2347 patients infected by Omicron BA.2 variant. Several inflammatory indicators were included and the derived neutrophil to lymphocyte ratio (dNLR) had a highest C-index in predicting the overall survival. These data suggested a simple but useful tool to predict the prognosis of COVID-19 patients, but did not point to interventions to modulate the dysregulated neutrophil function.

Zhang et al. performed a bioinformatic analysis for COVID-19-induced acute pancreatitis (AP), a relatively rare complication of SARS-CoV-2 infection using the scRNA-seq data in the Gene Expression Omnibus database. COVID-19 and AP shared some common transcriptional features such as the genes involved in “neutrophil degranulation”. A subset of mature activated neutrophils with high level of interferon-related genes were more enriched in COVID-19 patients, and numbers of this subset were correlated with the severity of AP.

In summary, the studies included in this topic provided an additional perspective on the importance of neutrophil death, and derangements in its expression, in the pathogenesis of critical illness, including sepsis, acute respiratory distress syndrome, ischemia reperfusion injury, COVID-19, and atherosclerosis.

## Author contributions

All authors listed have made a substantial, direct, and intellectual contribution to the work and approved it for publication.
